# Cross-cultural validation of the Educational Needs Assessment Tool in RA in 7 European countries

**DOI:** 10.1186/1471-2474-12-110

**Published:** 2011-05-24

**Authors:** Mwidimi Ndosi, Alan Tennant, Ulrika Bergsten, Marja Leena Kukkurainen, Pedro Machado, Jenny de la Torre-Aboki, Thea PM Vliet Vlieland, Heidi A Zangi, Jackie Hill

**Affiliations:** 1 Academic & Clinical Unit for Musculoskeletal Nursing (ACUMeN), Section of Musculoskeletal Disease, Leeds Institute of Molecular Medicine, University of Leeds, Leeds, UK; 2 Department of Rehabilitation Medicine, Section of Musculoskeletal Disease, Leeds Institute of Molecular Medicine, Faculty of Medicine and Health, The University of Leeds, Leeds, UK; 3 Research and Development Centre at Spenshult Hospital, Oskarström, Sweden; 4 The Finnish Society of Rheumatology Nurses, Helsinki, Finland; 5 Rheumatology Department, Coimbra University Hospital, Coimbra, Portugal; 6 Rheumatology Department, Alicante General and University Hospital, Alicante, Spain; 7 Department of Rheumatology and Department of Orthopaedics, Leiden University Medical Center, Leiden, The Netherlands; 8 National Resource Center for Rehabilitation in Rheumatology (NRRK), Diakonhjemmet Hospital, Oslo, Norway

**Keywords:** Cross-cultural validation, Outcome research, Patient education, Rasch analysis, Rheumatoid arthritis

## Abstract

**Background:**

The Educational Needs Assessment Tool (the ENAT) is a 39-item patient questionnaire originally developed in the UK to assess educational needs of patients with rheumatoid arthritis (RA). The objective of this study was to assess the cross-cultural validity of the ENAT in 7 European countries.

**Methods:**

The ENAT was translated into Dutch, Finnish, Norwegian, Portuguese, Spanish and Swedish versions by using Beaton's cross-cultural adaptation process, and was completed by a convenience sample of patients with RA in each country. The generated country-specific data were assessed for construct validity and were then pooled and assessed for cross-cultural invariance using Rasch analysis.

**Results:**

Individual country-specific analysis showed adequate fit to the Rasch model after adjustment for local dependency within domains. When data from the different countries were pooled, the 39 items deviated significantly from Rasch model's expectations (X^2 ^= 977.055, DF = 351, p = 0.000, PSI = 0.976). Again, most items within domains were found to be locally dependent, significantly affecting the fit. Consequently each domain was treated as a unit (i.e. testlet) and the ENAT was re-analysed as a seven-testlet scale resulting into a good fit to the Rasch model (X^2 ^= 71.909; DF = 63; p = 0.207, PSI = 0.951). A test of strict unidimensionality confirmed that all domains contributed to measuring a single construct. Cross-cultural non-invariance was discounted by splitting domains for DIF maintaining an excellent fit to the Rasch model. This allowed calibration of the ENAT into an interval scale.

**Conclusion:**

The ENAT is a simple tool, which is a valid measure of educational needs of people with RA. Adjustment for cross-cultural non-invariance is available if data from the 7 European countries are to be pooled or compared.

## Background

Rheumatoid arthritis (RA) is a chronic inflammatory, systemic disease largely affecting the synovium, which can lead to joint damage and bone destruction. It can affect the heart, lungs and eyes and causes severe disability, psychological distress and increased mortality [1,2]. Drug management aims to relieve symptoms and to modify the disease process. Despite new biologic treatments which are more efficacious and specific than other drug treatments [3,4], the patients' improvement in health status and quality of life may depend on their ability and willingness to adhere to all their therapies and undertake self-care activities. Patient education is the process by which patients are prepared for the latter important undertaking [5].

Patient education is recommended as an integral part of rheumatic diseases management [6,7] and ranges from supplying patient information leaflets to well-structured self-management programmes. However, systematic reviews have suggested that non-targeted education does not deliver long-term effects in RA patients [8,9]. Consequently recommendations have been made for patient education to be more patient-centred and tailored to address individuals' educational needs [10]. In order to plan effective patient-tailored education, clinicians need to assess patients' perceptions of their educational needs.

The Educational Needs Assessment Tool (the ENAT) is a patient-completed questionnaire designed to help patients with rheumatoid arthritis identify their educational needs. It was originally developed with patients and practitioners in the UK and it comprises 39 items grouped into 7 domains, namely: managing pain (6 items), movement (5 items), feelings (4 items), arthritis process (7 items), treatments (7 items), self-help measures (6 items) and support systems (4 items). The items are 5-category rating scales with descriptors: "not at all important", "a little important", "fairly important", "very important" and "extremely important". This gives a total score of educational needs ranging from 0-156. In the early development of the ENAT, two pilot studies were conducted among patients with various forms of arthritis [11]. The first one (with 20 patients) found the ENAT acceptable and easy to use and in the second (with 97 patients) the ENAT demonstrated a good test-retest reliability [11]. The original (English) ENAT was later completed by a sample of 125 patients with RA in the UK and its 7 domains demonstrated a good fit to the Rasch model indicating a good construct validity and supporting the unidimensionality of the scale [12].

Since patient education is a globally accepted part of treatment in RA and given the increasing need to undertake multinational studies, tools such as the ENAT also need to demonstrate a cross-cultural invariance (i.e. work in a consistent manner across countries) [13-15]. Thus cross-cultural validation of the ENAT would enable comparison of educational needs and data pooling across Europe. The objective of this research was to assess the cross-cultural validity of the ENAT in RA in 7 European countries.

## Methods

### Patients

This multicentre quantitative survey involved patients from the Netherlands, Finland, Norway, Portugal, Spain, Sweden and the UK. Each country was asked to provide at least 125 patients in order to achieve the minimum sample size recommended for Rasch analysis [16]. Apart from the Netherlands and Sweden, which used random sampling, all centres utilised convenience sampling methods to recruit patients from their rheumatology clinics, wards, day hospitals and databases. The inclusion criteria were age 18 or above, a positive diagnosis of RA and willingness to complete the ENAT. The exclusion criteria were (a) having any other rheumatic disease such as systemic lupus erythematosus, systemic sclerosis, psoriatic arthritis, ankylosing spondylitis and osteoarthritis, (b) inability to read or write and (c) those unwilling to participate. Participation was voluntary and each centre obtained an ethical approval from their local ethics committees.

### Measure

The original (English) ENAT was translated into the respective language versions by using the cross-cultural adaptation process recommended by Beaton et al [17]. The adaptation process involved 5 steps: 1) Initial translation - from the original (English) language to the target language 2) synthesis of the translations 3) back (blind) translation into the original (English) language 4) expert committee review which decides on equivalence between the source and target versions and 5) test of the pre-final version - testing the "adapted" version with 30 patients. This process was facilitated by an initial "set-up" meeting where the parameters for adaptation were considered and formalised. At this meeting, emphasis was placed upon the importance of the "conceptual" meaning of the statements in the ENAT.

The translated versions of the ENAT were given to patients in their respective countries to complete as postal surveys, or before their clinic consultations. The ENATs were anonymous but contained patients' demographical data such as gender, age, educational background and self-reported disease duration.

### Statistical analysis

Rasch analysis was used to assess the construct validity and the cross-cultural invariance of the ENAT [18]. Rasch analysis has been used in rheumatology in the development of new scales [19,20], to test the psychometric properties of existing scales [21,22] and for cross-cultural validation of patient outcome measures [14,23,24]. Since the Rasch model provides formal representation of fundamental measurement; fit to the model implies a criterion-related construct validity [25], objectivity [26], reliability [27] and statistical sufficiency [28]. Given fit to the Rasch model, ordinal data from an instrument can be converted into an interval scale, and parametric analytical methods can be used [29]. A more detailed description of the Rasch measurement model and its use in rheumatology is given elsewhere [30].

In the current study, data from individual countries were analysed separately and as pooled data, and subjected to Rasch analysis using RUMM2020 software [31]. Since the response format of the ENAT is polytomous, we utilised the Masters Partial Credit Model parameterisation [32]. Both the country-specific datasets and pooled data were assessed for fit, reliability and unidimensionality. The ideal fit values are given at the bottom of the Rasch analysis results table. In addition, the residual correlation matrix was examined and items that had a correlation of ± 0.3 were considered to display a local dependency [33]. These locally dependent items were combined into a "testlet". A testlet is defined as a subset of items that is treated as a measurement unit in test construction, administration and/or scoring [34]. The data, in the form of testlets were then tested for fit, unidimensionality and invariance. Strict unidimensionality was assessed by analysis of the principal components of the standardised residuals, the loadings upon which give rise to two sets of items to generate independent estimates, which are then compared using the independent t-test method suggested by Smith [35]. The reliability of the ENAT was assessed by Person Separation Index (PSI) which provides the estimate of the internal consistency of the scale using the logit value for each person as opposed to the raw score used in Cronbach's alpha [36].

The test of invariance (DIF analysis) was based on person factors such as gender, age, disease duration, educational background and country. To allow for comparisons, the continuous data (age and disease duration) were converted into categorical variables by splitting them at their country-specific medians. Education was categorised as basic (up to secondary education) or higher (high school - university) education. Item characteristic curves for each testlet were checked for any significant DIF with respect to any person factor. Since there were 7 countries, post-hoc analysis of cross-cultural DIF (Tukey test) was performed to ascertain cross-cultural DIF patterns. Subsequently the testlets affected with uniform DIF were "split" for DIF in order to adjust for the bias [37]. To avoid type I errors due to multiple testing, the *p*-values for fit statistics and DIF analyses were Bonferroni-adjusted to the alpha level (i.e. p = 0.05/number of tests carried out) [38].

## Results

### Sample characteristics

The sample of 1042 comprised 135 patients from Finland, 165 from the Netherlands, 137 from Norway, 123 from Portugal, 230 from Spain, 125 from Sweden and 125 from the UK. Their country-specific gender distribution, median age, median disease duration and educational background are summarised in Table 1.

**Table 1:** Sample characteristics by country

	Finland	The Netherlands	Norway	Portugal	Spain	Sweden	UK	Total
Sample size	135	165	137	123	230	125	125	1042

Percentage of females (%)	82.2	88.5	73.7	71.5	75.2	76.0	79.2	78.0

Median age (years)	54	67	57	52	58	61	58	

Median disease duration (years)	10	12	7	11	10	15	13	

Basic education (n)	53	29	105	67	111	50	105	473

Higher education (n)	81	134	32	47	104	73	17	488

### Individual country fit

Initially the data from each country was fitted to the Rasch model separately (Table 2). Local dependency was observed within each domain, and so the 39 items were grouped into 7 domains (testlets) and re-analysed. Fit to the Rasch model was then satisfied in each country including unidimensionality, with the exception of Portugal, where marginal multi-dimensionality was observed. An analysis of differential item functioning showed absence of bias for age, gender, educational level and disease duration across all countries except Spain, where two items (movement and feelings) showed bias for gender and disease duration.

**Table 2:** Rasch analysis results

		Item Fit Residual		Person Fit Residual		Chi Square Interaction		PSI		Independent T-Tests (CI)
			
	Analysis	Mean	SD	Mean	SD	Value (DF)	p		N	
UK	Analysis1 (initial)	0.34	1.686	-0.269	2.008	442.439 (351)	0.000	0.972	119	0.218 (0.179-0.258)
	
	Analysis 2 (Subtest)	0.541	0.699	-0.308	1.168	7.116 (7)	0.417	0.947	119	0.068 (0.028 - 0.107)

The Netherlands	Analysis1 (initial)	0.294	1.669	-0.393	2.285	130.721 (78)	0.000	0.967	163	0.215 (0.181 -0.249)
	
	Analysis 2 (Subtest)	0.433	1.365	-0.335	1.235	14.511 (14)	0.412	0.944	153	0.042 (0.006 - 0.078)

Finland	Analysis1 (initial)	0.190	1.611	-0.347	2.195	94.144 (39)	0.000	0.961	133	0.215 (0.178 - 0.253)
	
	Analysis 2 (Subtest)	0.333	1.037	-0.364	1.342	14.977 (14)	0.380	0.900	130	0.078 (0.040 - 0.116)

Norway	Analysis1 (initial)	0.171	1.438	-0.393	2.025	73.617 (39)	0.001	0.965	133	0.235 (0.198-0.272)
	
	Analysis 2 (Subtest)	0.542	0.873	-0.357	1.150	6.053 (7)	0.534	0.919	131	0.056 (0.018-0.094)

Portugal	Analysis1 (initial)	0.426	1.355	-0.515	2.624	56.025 (39)	0.038	0.985	114	0.211 (0.171-0.251)
	
	Analysis 2 (Subtest)	0.309	0.901	-0.449	1.337	2.625 (7)	0.917	0.975	114	0.105 (0.065 - 0.145)

Spain	Analysis1 (initial)	0.610	2.531	-0.351	2.485	781.797 (351)	0.000	0.981	187	0.214 (0.183 - 0.245)
	
	Analysis 2 (Subtest)	0.544	0.854	-0.375	1.293	17.450 (14)	0.233	0.963	186	0.022 (-0.010 - 0.053)

Sweden	Analysis1 (initial)	0.307	1.377	-0.248	2.032	93.612 (39)	0.000	0.972	119	0.303 (0.263 - 0.342)
	
	Analysis 2 (Subtest)	0.406	0.941	-0.322	1.146	5.409 (7)	0.610	0.944	118	0.061 (0.021 - 0.101)

Pooled	Analysis1 (initial)	1.018	4.014	-0.552	2.554	977.055 (351)	0.000	0.976	968	0.185 (0.171 - 0.198)
	
	Analysis 2 (Subtest)	0.847	1.475	-0.466	1.381	71.909 (63)	0.207	0.951	951	0.048 (0.034 - 0.063)

Expected values for a perfect model fit		0	1	0	1		> 0.05	> 0.85		0.05 (or Lower CI <0.05)

SD = Standard deviation, DF = Degrees of freedom, P = Χ^2 ^probability, (significant P = item misfit), PSI = Person

separation index, CI = Confidence intervals

### Pooled data

Initial Rasch analysis of the 39 items from the pooled data revealed significant deviations from the expectations of the Rasch model (X^2 ^= 977.055, DF = 351, p = 0.000) and a high reliability (PSI = 0.976) (Table 2). Item fit residuals had a mean of 1.018 (SD = 4.014) and person fit residuals had a mean of 0.552 (SD = 2.554). Nine items displayed fit residuals values outside the ± 2.5 expected range and a probability less than the Bonferroni adjusted α-value of 0.0013 indicating significant deviation from the model.

The patterns of the items' thresholds were examined and 5/39 items displayed borderline disordering. These items were: 1) Using acupuncture, ultrasound or hydrotherapy (pain) 2) Devices which would help me do practical things (Movement) 3) Why I am feeling down or depressed (Feelings) 4) How arthritis might affect my children or relatives (Arthritis process) 5) Times when I should call the doctor or nurse (Self-help measures). Examination of the category probability curves for the above items indicated the need to amalgamate two categories "a little important" and "fairly important" for all the five items. A trial rescoring improved the threshold ordering but the overall fit worsened, therefore the category structures of these items were not re-scored.

### Local dependency and unidimensionality

**Figure 1:**
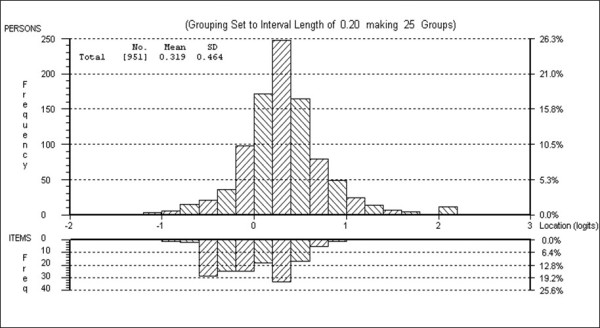
**Person-item threshold distribution**.

Examination of the person-item residual correlation matrix revealed that most domain-specific items were locally dependent and this was significantly affecting the fit to the model. All domain-specific items were then amalgamated into a testlet (each testlet corresponding to one ENAT domain) and the ENAT was then re-analysed as a seven-testlet scale which showed an acceptable fit to the Rasch model (X^2 ^= 71.909; DF = 63; p = 0.207) (Table 2 and Table 3). The strict unidimensionality test revealed the proportion of significant t-tests to be 0.048 (95%CI = 0.034 - 0.063) confirming its unidimensionality. The reliability of the final ENAT was excellent (PSI = 0.951). All analyses were thereafter based on the domain (testlet) scores. The person-item threshold distribution indicated that only a small proportion of the sample was above the range of the measurement indicating the ability of the ENAT to capture well the educational needs of patients (Figure 1).

**Table 3:** Item (testlet) fit

*Item*	*Location*	*SE*	*Fit residual*	*DF*	*Χ^2^*	*P*
Pain	0	0.010	0.176	743.47	3.748	0.927

Movement	0.014	0.010	-0.304	784.07	12.637	0.179

Feelings	0.097	0.011	3.760	775.61	14.563	0.104

Arthritis process	-0.139	0.009	-0.309	756.16	13.326	0.148

Treatments	-0.061	0.009	1.843	762.92	7.538	0.581

Self-help measures	-0.060	0.010	0.274	769.69	9.104	0.428

Support systems	0.149	0.012	0.490	773.07	10.992	0.276

SE = Standard error, DF = Degrees of freedom, Χ^2 ^= Chi Square statistic, P = Χ^2 ^probability (significant P = item misfit)

### Differential item functioning

Following fit to the Rasch model, DIF analysis on the pooled data revealed DIF by gender, age, disease duration, educational background and by country. However, apart from domain 3 (feelings) which displayed a non-uniform DIF by gender, all the DIF was uniform. Post-hoc analyses revealed that the cross-cultural DIF was the most significant contributing factor.

The Dutch data alone contributed to DIF by country on 4 testlets (pain, feelings, treatments and support). Splitting for DIF by country resolved all the cross-cultural DIF and most other sources of DIF. Testlet 2 (movement) continued to display uniform DIF by gender and by disease duration, while testlet 6 (self-help measures) had borderline DIF by educational background.

### Calibration of the ENAT into an interval scale

Following the adjustment for the cross-cultural DIF, the ENAT maintained a good fit to the Rasch model (X^2 ^= 138.311, DF = 162, p = 0.214) and an excellent reliability (PSI = 0.951). The ENAT domain raw scores were mapped against the corresponding Rasch transformed scores (in logits) and were linearly transformed to calibrate an interval-level scale of the same range (Table 4). A separate DIF-adjusted table was calibrated for the Dutch cohort (Table 5).

**Table 4:** Conversion of raw ENAT domain scores to Rasch-transformed values.

Raw Score	Rasch transformed score						
	**Pain**	**Movement**	**Feelings**	**Arthritis**	**Treatments**	**Self-help**	**Support**

0.0	0.0	0.0	0.0	0.0	0.0	0.0	0.0

1.0	2.7	1.9	1.8	2.1	2.2	1.5	1.8

2.0	4.5	3.1	3.1	3.6	3.6	2.7	3.0

3.0	5.6	4.1	4.0	4.6	4.6	3.6	4.0

4.0	6.6	4.8	4.8	5.4	5.6	4.3	4.8

5.0	7.3	5.6	5.7	6.2	6.3	5.0	5.5

6.0	8.1	6.2	6.3	6.9	7.1	5.5	6.1

7.0	8.7	6.8	7.0	7.5	7.8	6.1	6.8

8.0	9.3	7.4	7.7	8.2	8.4	6.8	7.5

9.0	9.9	8.1	8.3	8.9	9.1	7.5	8.1

10.0	10.5	8.7	9.0	9.5	9.8	8.2	8.9

11.0	11.1	9.3	9.7	10.3	10.4	9.1	9.6

12.0	11.6	10.0	10.5	11.2	11.3	10.2	10.4

13.0	12.2	10.7	11.4	12.0	12.1	11.3	11.3

14.0	12.8	11.5	12.5	13.0	13.1	12.4	12.3

15.0	13.4	12.3	13.9	13.9	14.1	13.5	13.9

16.0	14.1	13.1	16.0	15.1	14.9	14.4	16.0

17.0	14.6	14.3		15.9	15.9	15.3	

18.0	15.3	15.5		16.9	16.7	16.2	

19.0	16.1	17.4		17.7	17.4	17.1	

20.0	17.0	20.0		18.4	18.2	18.0	

21.0	17.9			19.2	19.0	18.9	

22.0	19.3			20.0	19.7	20.0	

23.0	21.1			20.8	20.5	21.7	

24.0	24.0			21.6	21.3	24.0	

25.0				22.6	22.3		

26.0				23.9	23.7		

27.0				25.6	25.3		

28.0				28.0	28.0		

**Table 5:** Conversion of raw ENAT domain scores to Rasch-transformed values for the Dutch dataset.

Raw Score	Rasch transformed score						
	**Pain**	**Movement**	**Feeling**	**Arthritis**	**Treatments**	**Self-help**	**Support**

0.0	0.0	0.0	0.0	0.0	0.0	0.0	0.0

1.0	3.3	1.9	1.0	2.1	2.1	1.4	1.7

2.0	5.5	3.2	1.9	3.6	3.7	2.6	2.9

3.0	7.0	4.2	2.6	4.6	4.6	3.4	3.8

4.0	8.2	4.9	3.2	5.4	5.5	4.2	4.5

5.0	9.2	5.6	3.7	6.0	6.2	4.9	5.2

6.0	10.1	6.3	4.3	6.8	6.9	5.5	5.9

7.0	10.9	6.9	4.9	7.5	7.6	6.0	6.5

8.0	11.7	7.4	5.4	8.1	8.3	6.6	7.3

9.0	12.4	8.2	6.0	8.8	8.9	7.3	7.9

10.0	12.9	8.8	6.7	9.4	9.6	8.0	8.7

11.0	13.5	9.4	7.5	10.3	10.1	8.9	9.5

12.0	14.0	10.1	8.5	11.1	10.8	10.0	10.3

13.0	14.6	10.9	9.7	11.9	11.7	11.2	11.2

14.0	15.0	11.6	11.1	12.9	12.4	12.4	12.4

15.0	15.6	12.5	13.2	13.8	13.3	13.4	13.9

16.0	16.1	13.3	16.0	15.0	14.2	14.3	16.0

17.0	16.6	14.3		15.8	15.1	15.3	

18.0	17.1	15.7		16.8	16.0	16.1	

19.0	17.7	17.5		17.6	16.7	17.0	

20.0	18.4	20.0		18.4	17.6	17.9	

21.0	19.1			19.2	18.3	18.8	

22.0	20.2			19.9	19.1	20.0	

23.0	21.6			20.7	20.0	21.7	

24.0	24.0			21.5	20.9	24.0	

25.0				22.6	22.0		

26.0				23.8	23.4		

27.0				25.6	25.4		

28.0				28.0	28.0		

## Discussion

This study has demonstrated that the ENAT satisfies Rasch model expectation in seven countries, with the possible exception of marginal multidimensionality in Portugal. The ENAT has been shown to be largely invariant by age, gender, educational level and disease duration with each country. When data were pooled, some DIF manifested, but was largely driven by country-specific DIF. When examined, most DIF was shown to cancel, but country DIF remained. Consequently when data are pooled across countries, adjustment must be made to accommodate the potential bias. Following such an adjustment, an interval scale transformation can be made, giving a raw-score interval metric table for general use.

A number of issues have arisen from this work. The breach of the local independence assumption has been shown to drive misfit to the Rasch model. This can be accommodated through the testlet design. This deals with the perennial problem of the tension between the clinimetric needs and the psychometric requirements of a measure when items that have clinical relevance are removed from the scale [39-41]. Leaving the items in the scale is advantageous in that the items may inform practitioners about educational needs at the finer level, while grouping them into testlets effectively accounts for the local dependence so satisfying the psychometric requirements [42].

The second issue is the implication of the lack of invariance across countries for pooling data in international studies. DIF analysis revealed that the cross-cultural DIF was responsible for most of the non-invariance in the data. Cross-cultural adjustment involved splitting the DIF-affected items by country, producing a scale with both etic (culturally-general) and emic (culturally-specific) items. This permits the scale to be culturally relevant while permitting comparisons across cultures and languages on the basis of the common etic items. Once the cross-cultural invariance was adjusted, the overall DIF improved including resolution of the non-uniform DIF by gender. When cross-cultural comparisons are to be performed, a separate DIF-adjusted conversion table for the emic data will need to be used. However, it should be stated that the magnitude of the observed DIF was only marginal, in that the maximum difference of location across countries within any educational need level (class interval) was only 0.18 logits. This suggests that the sample size of the pooled data was driving the statistical significance, and that the observed magnitude of DIF is below the level considered to be associated with measurement error [43].

As such, the ENAT can be used as a routine clinical checklist or as a research (or an audit) tool. In the former use, the clinician can use the ENAT as a simple checklist to assess perceived educational needs of patients before a clinic consultation. In this context, the perceived priority needs can be determined by looking at the completed ENAT without the need for scoring. In the latter use (measurement context) where the underlying latent construct of "educational need" is important, the Rasch-transformed scores give a common metric across all domains for comparative purposes. All domains contribute to measuring a single construct; thus adding up the domain scores is a sufficient statistic for estimating patients' educational needs (range = 0 to 156).

While the ENAT provides patients' perceived education needs, the health professional may know more about current treatment options and guidelines such as treat-to-target recommendations [44] which are beneficial to the patients. Having assessed patient's perceived educational priorities using the ENAT, the health professional can then discuss the needs arising with the patient, and provide more information about the available treatment goals and options in order to facilitate patient participation in their management. The main limitation of this study is that the ENAT is a self-completed questionnaire and consequently it did not reach the population of patients who cannot read and write. Further investigation of the marginal multidimensionality in Portugal would also be required. The ENAT can be obtained by contacting the corresponding author.

## Conclusion

While Patient education is recommended as an integral part of rheumatic diseases management, [6,7] knowing what aspect of education may be required by a patient at any specific point of their treatment is an essential prerequisite to ensure that such needs are met. The ENAT offers a simple tool to help professionals judge what is required. It satisfied the strictest standards of measurement in all but Portugal, where marginal multidimensionality was observed, and can offer interval scaling when required. The scale can be used with confidence within the countries studied, but if data are to be pooled, then this will require adjustment within the framework of the Rasch measurement model, so providing the ability to compare educational needs across Europe.

## Competing interests

The authors declare that they have no competing interests.

## Authors' contributions

JH & AT designed the study. JH, UB, JT, PM, MLK. TPMV & HZ collected the data. MN & AT undertook the statistical analysis. MN, AT & JH interpreted the results. All authors participated in the preparation of the manuscript, read and approved the final version.

## Pre-publication history

The pre-publication history for this paper can be accessed here:

http://www.biomedcentral.com/1471-2474/12/110/prepub
